# Ten-year follow-up of a prospective trial for the targeted therapy of gastric cancer with the human monoclonal antibody PAT-SC1

**DOI:** 10.3892/or.2014.2987

**Published:** 2014-01-20

**Authors:** FRANK HENSEL, WOLFGANG TIMMERMANN, BURKHARD H.A. VON RAHDEN, ANDREAS ROSENWALD, STEPHANIE BRÄNDLEIN, BERTRAM ILLERT

**Affiliations:** 1 Patrys GmbH, Würzburg, Germany; 2 Allgemeines Krankenhaus, Hagen, Germany; 3 Department of General, Visceral, Vascular and Pediatric Surgery, University of Würzburg, Würzburg, Germany; 4 Institute of Pathology, University Hospital, University of Würzburg, Würzburg, Germany; 5 Sana Kliniken Ostholstein GmbH, Eutin, Germany

**Keywords:** gastric cancer, antibody therapy, monoclonal antibody, PAT-SC1, prospective trial, gastrectomy, D2-lymphadenectomy, radical surgery

## Abstract

The fully human monoclonal antibody PAT-SC1 is specific for an isoform of CD55 (decay-accelerating factor) designated CD55^PAT-SC1^. This antigen is expressed in the majority (80%) of gastric cancers (GCs), and the antibody induces tumour cell-specific apoptosis *in vitro* as well as *in vivo*. PAT-SC1, therefore, has been deemed promising as a therapeutic agent. Here, we describe the results of an academic clinical study performed in a neoadjuvant setting with resectable GC patients. Patients undergoing treatment for GC between 1997 and 2001 were tested for CD55^PAT-SC1^ expression. Fifty-one resectable patients that tested positively received a single administration of 20 mg PAT-SC1 48 h prior to surgery. They underwent standard surgery with either subtotal or total gastrectomy with bursectomy, omentectomy and a modified D2-lymphadenectomy, aimed at R0 resection. Primary endpoints of the present study were to evaluate side-effects of the PAT-SC1 antibody treatment and to evaluate histopathological effects such as tumour regression and induction of apoptosis. Long-term survival was a secondary endpoint. Administration of PAT-SC1 appeared safe with only reversible side-effects according to WHO grade I and II. Despite the low-dose of the antibody, 81.6% of the patients showed signs of increased apoptosis within the primary tumour and 60% showed signs of tumour cell regression. Comparison of the 10-year survival rates of the R0-resected CD55^PAT-SC1^-positive patients treated with the PAT-SC1 antibody with a historical collective of R0-resected CD55^PAT-SC1^-positive patients not treated with PAT-SC1 indicated a survival benefit in the treated patients. Furthermore, comparison of the patient survival of CD55^PAT-SC1^-positive vs. CD55^PAT-SC1^-negative groups suggested that CD55^PAT-SC1^ antigen expression is an independent predictor of poor survival in a Cox regression analysis. Antibody PAT-SC1 may be a useful additive therapeutic agent in the treatment of patients with CD55^PAT-SC1^-expressing GCs. In combination with radical standard surgery, PAT-SC1 given as an adjuvant or neoadjuvant immunotherapeutic agent induces apoptosis in tumour cells which may improve survival of these patients. Because of the human origin and its specific binding to the CD55^PAT-SC1^ antigen, PAT-SC1 was well tolerated in this trial.

## Introduction

Gastric cancer (GC) constitutes the second major cause of cancer-related mortality worldwide (1). The poor prognosis of this disease, including its high relapse rate even after curative resection and a high disease related mortality, has not been substantially improved in recent decades.

Complete surgical resection is still the standard treatment for all patients with resectable GC; however, regional and less common systemic recurrence remain highly problematic. Although the role of extended lymphadenectomy remains controversial, it is still accepted that a modified D2-lymphadenectomy is reasonable, due to its beneficial role for a subgroup of patients (2–4). Yet, even among patients undergoing gastrectomy with curative intent, the 5-year survival rates are disappointingly low at 25 to 30% due to locoregional relapse and distant metastases (5).

These circumstances have led to various adjuvant and neoadjuvant protocols. Although a beneficial role is attributed to adjuvant therapies, these are associated with severe side-effects often resulting in premature termination of a protocol (6,7).

In contrast to these established procedures, the idea of tumour-specific therapy is captivating. Major advances have been achieved in the field of biological-based cancer therapies in the last decades. Some of the recently approved targeted therapeutics are currently being evaluated for the treatment of GC (8,9).

The PAT-SC1 antibody described here was isolated with the aid of human hybridoma technology from a GC patient (10). This fully human IgM antibody reacts with a cancer-specific isoform of CD55 decay-accelerating factor (DAF), subsequently named CD55^PAT-SC1^ (11). In previous studies the antigen was reported to be expressed in ~70% of diffuse-type gastric carcinoma and in 25% of intestinal-type according to the Lauren classification (12). More recent studies have demonstrated the expression of CD55^PAT-SC1^ in ~80% of patients across different ethnic groups (unpublished data). Therefore, PAT-SC1 has a much broader potential than other targeted therapies which are currently under evaluation for the treatment of GC. One such therapy, trastuzumab, was found to react with only 22.9% of advanced GCs (13). PAT-SC1 shows no cross-reactivity with adult normal tissues, but some cross-reactivity with embryonal tissue, indicating that the antigen may be of oncofetal origin (10,14).

Upon binding to CD55^PAT-SC1^, the antibody was found to induce apoptosis of stomach carcinoma cells *in vitro* (14) and *in vivo* and showed tumour-suppressing activity in xenograft animal models (15). The apoptotic effect was not only noted in the primary tumour but in disseminated tumour cells as well (16). Disseminated tumour cells in the blood of patients with GC are an independent predictive marker of poor prognosis (17).

Based on these promising results, an academic single-dose clinical study in patients with primary GC was initiated in 1997 with the primary aim to establish the safety of PAT-SC1 in humans and to confirm the ability of the PAT-SC1 antibody to induce tumour-specific apoptosis in a neoadjuvant setting. The study was conducted in 51 patients with positively proven expression of the CD55^PAT-SC1^ antigen. In addition to the safety and the histopathological effect of the antibody, the 10-year survival of the R0-resected patients was investigated and compared to a historical control group.

In addition, in a historical patient group, we analysed whether the expression of the PAT-SC1 antigen CD55^PAT-SC1^ is a prognostic factor for cancer-related survival.

## Patients and methods

### Antibody production and purification

The antibody was produced in miniPERM bioreactors and purified by a two-step purification scheme as outlined previously (18).

### Clinical protocol

In a prospective series from July 1997 to January 2001, patients with primary GC were tested for expression of CD55^PAT-SC1^. Preoperative biopsies (obtained endoscopically) from the cancer were stained immunohistochemically with the PAT-SC1 antibody according to the protocol published by Vollmers *et al* (12). In case of a positive reaction of the antibody with the tumour, the patient was defined as being CD55^PAT-SC1^-positive. Forty-eight hours prior to the surgical treatment, the patients were administered intravenously a single dose of 20 mg PAT-SC1 diluted in 500 ml infusion solution over 4 h. During the infusion, patients were placed on the intermediate care unit for the monitoring of vital parameters. All patients provided written informed consent for the preoperative antibody treatment. The study protocol was approved by the Ethics Committee of the University of Würzburg.

### Surgical procedure

For radical resection (R0) according to the Union Internationale Contre le Cancer (UICC) 1997, a total gastrectomy with a modified D2-lymphadenectomy according to the site of the tumour was performed. Lymphadenectomy included compartment I (lymph nodes along the greater and lesser curvature) and compartment II dependent on the site of the tumour. The lymph nodes on the upper margin of the pancreas and within the hilus of the spleen were removed only when the primary tumour affected the corpus or left sided margin of the stomach. If the tumour was located in the distal part of the stomach, lymph nodes within the hepatoduodenal ligament and paraaortic were dissected as well (compartment III/IV). The tail of the pancreas and the spleen were resected only when directly involved by the tumour.

### Study population

Fifty-one patients with primary carcinoma of the stomach expressing CD55^PAT-SC1^ were included in the study and were consecutively treated with the human monoclonal antibody PAT-SC1. The details of the patient population are summarized in Table I (group 3).

### Historical data collection for the patients with GC for retrospective analysis of the expression of the PAT-SC1 antigen as a prognostic marker

To prove if the expression of CD55^PAT-SC1^ is of prognostic value, historical patient data from the University Hospital Würzburg were evaluated. For this comparison, data were included from patients who underwent radical gastrectomy and lymphadenectomy due to GC prior to 1997. Surgery was performed using the same methodology as in the study group (group 3/Table I). No adjuvant or neoadjuvant treatment was performed. Antigen expression of these patients was determined from paraffin-embedded tumour material, retrospectively. One hundred twenty-six patients were included: 93 were positive (group 1/Table I) and 33 were negative (group 2/Table I) for CD55^PAT-SC1^ expression. Both groups (CD55^PAT-SC1^-positive and CD55^PAT-SC1^-negative) were comparable in regards to UICC stage (p=0.8121 Chi-square test).

### Tumour regression

A semi-quantitative analysis was performed by two independent experts, who microscopically evaluated three fields from each tumour specimen and graded them on a scale of 1 to 3: 1, low; 2, frequent; 3, high.

The values for the three fields were added together, and then subtracted from the total score obtained from the three fields of the biopsies taken before PAT-SC1 treatment. If the difference was <2, tumour regression was graded as 0; if the difference was between 2 and 3, tumour regression was graded as (+); if the difference was ≥4, tumour regression was rated as (++).

A consensus value was found between the experts as the final scoring of tumour regression.

### Analysis of apoptosis

Staining for apoptosis was performed as described previously (18). A semi-quantitative analysis was performed by two independent experts, who evaluated via microscopy three fields from the tumour specimens and graded them in comparison to the biopsy taken before the treatment. The scoring range from no apoptosis (−) to (+++) using a Tunel assay: (−), negative, similar to biopsy taken prior to PAT-SC1 treatment; (+), up to 30% more apoptotic cells; (++), up to 60% more apoptotic cells and (+++), 60 to 100% apoptotic cells. As a reference, DNase-induced apoptosis was determined to be 100%.

### Follow-up

All patient data were followed up with the tumour registry of the University of Würzburg. Patients with curative resection were followed up within the first and second year after surgery every three months in our outpatient department or at the general practitioner’s office to perform follow-up evaluation. The follow-up included patient history, a physical examination and blood tests (full blood count, electrolyte values, blood urea nitrogen, liver function tests and tumour markers). Endoscopy of the upper GI-tract was performed 12 months after surgery. After the second year, the patients were followed up once each year for routine examination. Further examinations were performed according to changes in the clinical status of the patient. The follow-up after palliative resection was performed according to the clinical status of each patient individually.

### Data evaluation and statistical analysis

Patient data, side-effects, response to antibody treatment (apoptosis of tumour cells, regression of tumour), postoperative course, histopathological evaluation (staging and grading according to TNM and UICC) and follow-up were recorded. Comparison of UICC stages in the study group and the comparison group was performed with the two group Chi-square test. The survival rate was determined by Kaplan-Meier analysis. Differences between survival curves were calculated with the log-rank test. p<0.05 was considered to indicate a statistically significant result.

## Results

### Patient characteristics

There was no 30-day mortality. The follow-up period was carried out for a minimum of 120 months. The evaluation described here was focused on the patient group undergoing curative (R0) resection. Of this group, 16 patients died of tumour-related causes, 18 patients were alive at the time of evaluation and there was one non-tumour-related death.

### Side-effects due to the antibody therapy

The infusion with the PAT-SC1 antibody was generally well-tolerated. Nineteen (37.3%) patients exhibited a total of 35 adverse events, 32 of grade 1, and 3 of grade 2 (Table II). More severe side-effects of grade 3 or 4 were not observed. One patient had a short period of shaking chill, which led to the termination of the PAT-SC1 infusion after injection of 15 mg. One patient had an early termination of the infusion due to an anaphylactic reaction and hypotension. All other patients received the scheduled amount with a median dose of 20 mg (2 patients 10 mg, 44 patients 20 mg, 3 patients 30 mg).

### Apoptosis and regression

**Figure 1:**
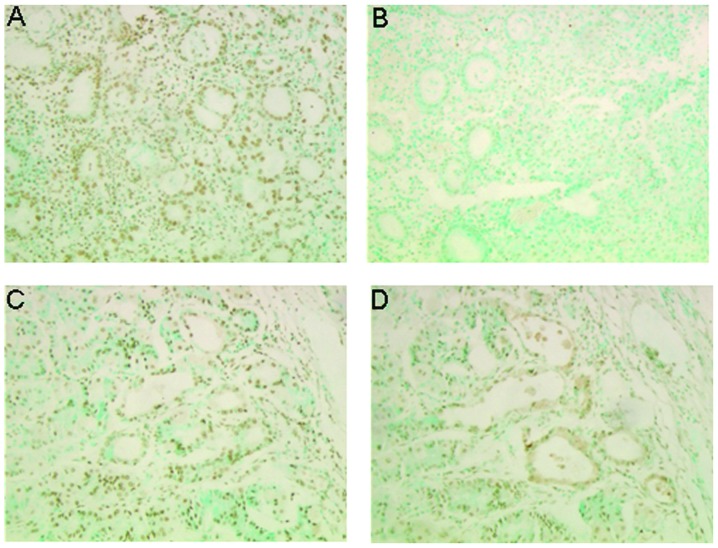
Analysis of the apoptotic activity of the PAT-SC1 antibody *in vivo*. (A and B) A pre-treatment biopsy and (C and D) a post-treatment tumour sample of a stomach carcinoma patient were investigated for PAT-SC1-induced apoptosis using the Klenow FragEL DNA fragmentation kit (Oncogene, Boston, MA, USA). (A and C) In the positive controls, all cell nuclei are stained due to treatment with an endonuclease. (B and D) In the images (right panels) only the nuclei of apoptotic stomach tumour cells are stained. (B) The pre-treatment biopsy shows no apoptotic activity. (D) In the post-treatment tumour sample an increase in apoptotic tumour cells after PAT-SC1 treatment is noted.

The primary endpoint was the measurement and comparison of apoptosis in the resected tumour compared to the pre-treatment biopsy by a Klenow nicked-end DNA labeling IHC assay on paraffin-embedded tissue sections. In Fig. 1 an example of analysis of apoptosis is shown. The pre-treatment biopsy showed no apoptotic cells whereas an increase in the amount of apoptotic cells was demonstrated in the post-treatment tumour sample. A high (++) (n=21) or moderate (+) (n=18) increase in apoptosis after antibody treatment was noted in 39 out of 51 (78%) patients, and in 9 (18%) patients no increase in apoptosis (−) was noted (Table III). In 2 (4%) patients, the analysis could not be performed due to the small amount of tumour cells in the samples, and 1 (2%) patient was not included due to another tumour resection prior to the gastrectomy. There was no statistically significant difference in the degree of apoptosis and patient survival (p=0.49).

Twenty-six patients (52%) showed a high (++) (n=15) or moderate (+) (n=11) increase in tumour regression after antibody treatment and 18 (36%) patients showed no regression (−) after PAT-SC1 therapy (Table III). In 6 (12%) patients, the analysis was not performed due to technical problems. Patients with a high degree (++) of regression had a statistically significant better survival (p=0.0214) compared to the survival of patients with moderate or no tumour regression.

### CD55^PAT-SC1^ expression as a diagnostic marker

**Figure 2:**
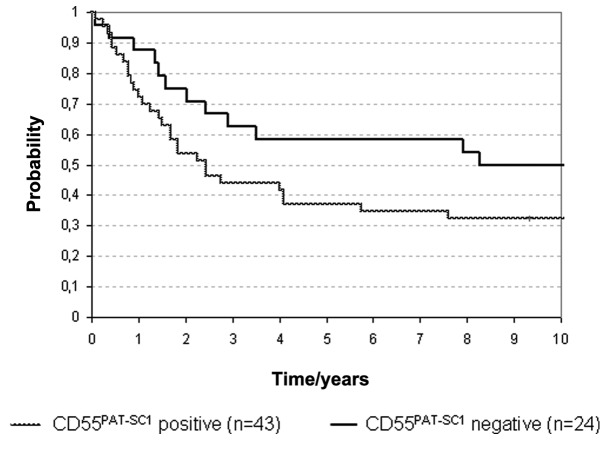
Ten-year overall survival rates of the R0-resected patients as assessed by Kaplan-Meier analysis. Kaplan-Meier-curve of R0-resected, CD55^PAT-SC1^-expressing gastric cancer patients (dashed line) vs. R0-resected gastric cancer patients which did not express CD55^PAT-SC1^ (black line). Gastric cancer patients that did not express CD55^PAT-SC1^ tended to have a prolonged rate when compared to those expressing CD55^PAT-SC1^ (p=0.237).

To investigate whether the expression of CD55^PAT-SC1^ has any influence on the prognosis of GC patients, a historical patient population (prior to 1997) was tested for the expression of the CD55^PAT-SC1^ by immunohistochemistry, and the correlation with the survival of the R0 resected patients was assessed (Fig. 2). The survival rates of the 10-year follow-up were determined for 43 CD55^PAT-SC1^-positive patients (group 1) and 24 CD55^PAT-SC1^-negative patients (group 2).

The data indicate that the expression of CD55^PAT-SC1^ had a trend toward a correlation with poorer survival but did not reach statistical significance with the number of patients studied (p=0.227).

### Distribution of the tumour stages

**Figure 3:**
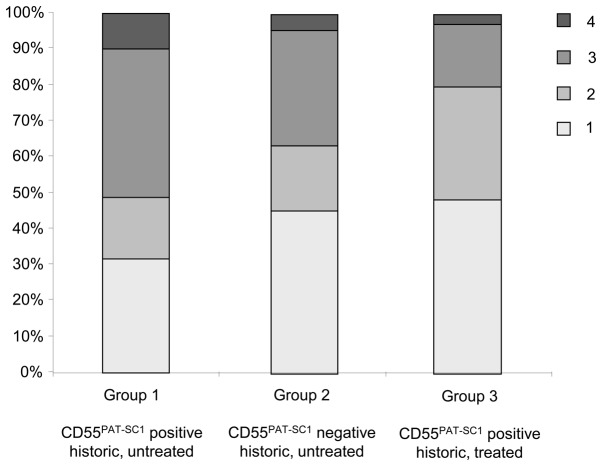
Distribution of the UICC stages within the different groups evaluated. Distribution of UICC stages 1 to 4 of the R0-resected patients. UICC, Union Internationale Contre le Cancer.

To verify the comparability of the study groups, the distribution of the tumour stages was evaluated (Fig. 3). The staging was available for 41 of the 43 (95.3%) CD55^PAT-SC1^-positive and untreated patients (group 1), 22 of the 24 (91.6%) CD55^PAT-SC1^-negative and untreated patients (group 2) and all (n=35, 100%) of the CD55^PAT-SC1^-positive treated patients (group 3).

The data indicated that the tumour stages in the CD55^PAT-SC1^-positive untreated patients were significantly higher (51% stage 3 and 4) than the stages in the CD55^PAT-SC1^-positive but treated patient group (20% stage 3 and 4). There were no other inclusion criteria other than operability for inclusion into the PAT-SC1-treated group.

### Results of retrospective analysis

**Figure 4:**
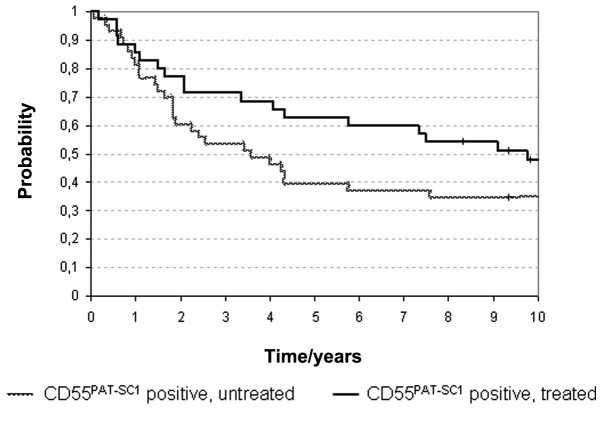
Ten-year overall survival rates of the R0-resected patients with or without PAT-SC1 antibody treatment as assessed by Kaplan-Meier analysis. Kaplan-Meier curves of R0-resected, CD55^PAT-SC1^-expressing gastric cancer patients following PAT-SC1 treatment (black line) vs. R0-resected. CD55^PAT-SC1^-expressing untreated gastric cancer patients (dashed line). PAT-SC1-treated patients tended to have a survival benefit when compared to the untreated patients (p=0.219).

Retrospective analysis of the 10-year survival of CD55^PAT-SC1^-positive, untreated patients (group 1) vs. the CD55^PAT-SC1^-positive, treated group revealed a survival benefit (Fig. 4). While in the group 1 only 15 out of 43 (34.9%) patients were alive, and in group 3 (CD55^PAT-SC1^-positive, treated), 16 out of 35 (45.7%) patients were alive 10 years after antibody treatment and curative tumour resection.

## Discussion

This is the first long-term follow-up of a clinical trial assessing an additive treatment for stomach cancer consisting of a fully human IgM antibody directed specifically against the tumour-specific CD55^PAT-SC1^ variant of CD55. In comparison to radiation or chemotherapy, the effect of PAT-SC1 is highly specific for susceptible tumour cells that express the CD55^PAT-SC1^. PAT-SC1 also has broader reactivity for GCs than other targeted therapeutics currently under investigation.

The present study showed three main results. i) The previously described effects of the PAT-SC1 antibody *in vitro* and *in vivo*, which included induction of apoptosis in tumour cells and tumour regression, were confirmed in the majority of PAT-SC1-treated patients. ii) The safety and tolerability of the human antibody PAT-SC1 was excellent with no major side-effects following antibody treatment. iii) The survival rate of R0-resected, CD55^PAT-SC1^-positive, PAT-SC1 antibody-treated patients after 10 years was prolonged compared to a historical control group of CD55^PAT-SC1^-positive patients with R0-resection not treated with the PAT-SC1 antibody.

### Apoptosis and tumour regression due to PAT-SC1 antibody treatment

PAT-SC1 is a natural human IgM antibody with high specificity to tumour cells of GC. Following the binding of the antibody to the CD55^PAT-SC1^ antigen on tumour cells *in vitro*, the antibody triggers highly specific apoptosis (14). GC xenograft models with PAT-SC1 showed that tumour cell-specific apoptosis can also be induced *in vivo*, and a tumour reduction of up to 80% was achieved (15,16).

The present study demonstrated that even with low doses of the antibody, an induction of tumour regression and apoptosis of gastric tumour cells in patients, prior to standard surgical resection, were induced. We compared preoperative biopsies from the CD55^PAT-SC1^-positive tumours before antibody therapy and after antibody therapy and tumour resection. Despite the small dose of the antibody (mean, 20 mg/patient) an increase in apoptosis was observed in 39 of the 48 (81%) patients evaluated. Tumour regression was observed in 26 of the 44 (59.1%) patients, thus confirming the previously described effects *in vitro* and *in vivo* animal models. Tumour regression studies have shown PAT-SC1 to be of prognostic value (19), and the finding of increased tumour regression after antibody treatment and increased 10-year survival rate indicates the effectiveness of the antibody.

### Tolerability of PAT-SC1

In the present study the treatment with the PAT-SC1 antibody did not cause severe side-effects. The observed side-effects were all of a minor nature not exceeding grade II (according to the WHO, Common Toxicity Criteria). While the relatively low dose of the PAT-SC1 antibody tested in this trial may be one reason for this finding, one can speculate that the human origin and germline configuration of the PAT-SC1 antibody contributes to the good tolerability of the antibody. Furthermore, the antigen, CD55^PAT-SC1^, to date, has been only detected on cancer tissues, therefore decreasing the chance of unwanted side-effects on healthy tissues as noted for other targeted therapeutics. Recent studies with approved antibodies for GC showed more severe side-effects such as gastric perforation or thromboembolic events as observed in therapy with bevacizumab (20) Additional analysis revealed that the application of apoptosis-inducing antibody PAT-SC1 prior to surgery of gastric tumours had a mild if any effect on the immune system. Therefore, from an immunological point of view, the treatment with this monoclonal antibody is extremely safe (21).

### Survival analysis and role of minimal residual disease

Although overall survival was not an endpoint of our study, we evaluated the 10-year survival of the PAT-SC1-treated patients vs. a historical control group. The data revealed that despite the low dose of PAT-SC1, a benefit in the 10-year survival rate was observed. We found that the survival of CD55^PAT-SC1^-expressing GC patients was increased after neoadjuvant treatment with PAT-SC1 compared to a historic control group of CD55^PAT-SC1^-positive patients (49 vs. 35%) even after an observation period of 10 years. This may be regarded as further evidence that neoadjuvant or additive therapies may improve patient survival after radical resection of GC. In a recent meta-analysis, a slight benefit in postoperative survival was described for patients with additional chemotherapy. The intergroup study reported a significant 15% survival benefit for patients receiving postoperative radiochemotherapy compared to those in a surgery only group (22).

Despite the fact that in the present study the patients of the different groups were not randomized but selected solely on the basis of CD55^PAT-SC1^ expression and despite the small number of patients included, the biological effect of the PAT-SC1 treatment cannot be ignored. Notably, in our treatment group only minor side-effects were observed whereas in the intergroup study, the toxicity of the adjuvant protocol was high (WHO grade III in 6% of patients). An influence of the CD55^PAT-SC1^ status on the prolonged survival of our patients can be ruled out as our data indicate that the expression of CD55^PAT-SC1^ is a negative prognostic factor. In our retrospective data analysis, patients with tumours not expressing the CD55^PAT-SC1^ had a better survival rate than those with tumours expressing the antigen and not receiving antibody therapy. Our favoured hypothesis for the therapeutical effect of PAT-SC1 as additive therapy in addition to radical surgery is a reduction in disseminated tumour cells (DTCs). DTCs are a sign of minimal residual disease (MRD), and confirmation of DTCs is discussed as a reason for relapse and occurrence of peritoneal or distant metastases (23,24). DTCs have been detected in many patients with different types of solid tumours (25). The presence of DTCs and MRD in cancer patients is predictive of a poor clinical outcome (24). The effective elimination of DTCs was found to result in a better prognosis in breast cancer patients (24). In a study by our group we identified DTCs in the venous blood of GC patients as an independent marker of poor prognosis (17).

As long as DTCs are in a non-proliferating status of dormancy, most adjuvant agents fail to eliminate DTCs (26). Antibodies such as PAT-SC1 which induce apoptosis in cancer cells irrespective of their status of proliferation may represent a much more effective tool for reducing MRD and thus improving patient prognosis if local clearance of the tumour has been achieved by radical surgery. Furthermore, we showed that DTCs can be detected in an animal model with GC (27) and that therapy of the animals with PAT-SC1 reduced the DTCs (16). Expression of CD55^PAT-SC1^ was detectable in positive lymph nodes, distant metastases and tumour cells of the peritoneal cavity in patients with GC (18). For this reason, the PAT-SC1 antibody may be highly effective for all forms of MRD.

In conclusion, the human IgM antibody PAT-SC1 induces highly specific apoptosis in tumour cells expressing the tumour-specific variant of CD55. In contrast to other therapeutic antibodies against solid tumours, the PAT-SC1 antibody is well tolerated by patients. The expression of CD55^PAT-SC1^ can easily be determined in preoperative biopsies, which offers a novel patient- and tumour-specific neoadjuvant therapy for GC. Together with radical surgical treatment, a single preoperatively administered dose of PAT-SC1 may improve patient survival possibly through reduction of MRD. However, given the disparity of the stages between the treatment and the historical control groups it is not possible to draw definitive conclusions in respect to the survival benefit. To verify this, a randomized trial is necessary which is justified based on the survival difference observed along with the histopathological data. Given that there is an increasing amount of data indicating that IgM antibodies are suitable therapeutic agents, further research is needed in order to advance this class of antibodies for use in the clinic.

## Figures and Tables

**Table I:** Demographic features and clinical staging of the groups used for the evaluation of PAT-SC1 effect and PAT-SC1 antigen expression as a diagnostic/prognostic marker.

	Group 1 CD55^PAT-SC1^-positive (prior to 1997) (n=93)	Group 2 CD55^PAT-SC1^-negative (prior to 1997) (n=33)	Group 3 CD55^PAT-SC1^-positive (after 1997) (n=51)
Age (years) mean ± SD	63.7±11.5	64.4±12.0	62.6±12.6
Gender n (%)			
Female	32 (34.4)	11 (33,3)	26 (51,0)
Male	61 (65.6)	22 (66.7)	25 (49.0)
Histological stage (%)			
Adenocarcinoma (intestinal type)	47 (50.5)	18 (54.5)	9 (17.6)
Signet ring cell carcinoma (diffuse type)	43 (46.2)	15 (45.5)	36 (70.6)
Other	3 (3.2)		6 (11.8)
UICC staging (%)			
1A	9 (9.7)	5 (15.2)	10 (19.6)
1B	8 (8.6)	5 (15.2)	10 (19.6)
2	15 (16.1)	7 (21.2)	11 (21.6)
3A	20 (21.5)	8 (24.2)	3 (5.9)
3B	10 (10.8)	2 (6.1)	4 (7.8)
4 29	(31.2)	5 (15.2)	12 (23.5)
X	2 (2.2)	1 (3.0)	1 (2.0)
Residual tumour classification (%)			
R0	53 (57.0)	26 (78.8)	35 (70.0)
R1	11 (11.8)	3 (9.1)	2 (4.0)
R2	29 (31.2)	4 (12.1)	13 (26.0)

UICC, Union Internationale Contre le Cancer.

**Table II:** Side-effects observed during PAT-SC1 treatment.

System Organ Class	Grade 1	Grade 2	Grade 3	Grade 4	All grades
Preferred Term	n (%)	n (%)	n (%)	n (%)	n (%)
Cardiac disorders	4 (7.8)				4 (7.8)
Bradycardia NOS	2 (3.9)				2 (3.9)
Tachycardia NOS	2 (3.9)				2 (3.9)
Gastrointestinal disorders	1 (2.0)				1 (2.0)
Nausea	1 (2.0)				1 (2.0)
General disorders and administration side conditions	13 (25.5)	1 (2.0)			14 (27.5)
Application site cold feeling	4 (7.8)	1 (2.0)			4 (7.8)
Pyrexia	7 (13.7)				8 (15.7)
Rigors	2 (3.9)				2 (3.9)
Immune system disorders	1 (2.0)	1 (2.0)			2 (3.9)
Anaphylactic reaction	1 (2.0)	1 (2.0)			2 (3.9)
Musculoskeletal and connective tissue disorders	3 (5.9)				3 (5.9)
Arthralgia	1 (2.0)				1 (2.0)
Back pain	1 (2.0)				1 (2.0)
Groin pain	1 (2.0)				1 (2.0)
Nervous system disorders	4 (7.8)				4 (7.8)
Dizziness	1 (2.0)				1 (2.0)
Headache	3 (5.9)				3 (5.9)
Vascular disorders	6 (11.8)	1 (2.0)			7 (13.7)
Cyanosis peripheral	1 (2.0)				1 (2.0)
Hypertension NOS	2 (3.9)				2 (3.9)
Hypotension NOS	3 (5.9)	1 (2.0)			4 (7.8)

Note, no grade 3 or 4 side-effects were observed, NOS, not otherwise specified.

**Table III:** Residual tumour classification and histological examination of the primary tumours for occurrence of apoptosis and tumour regression after PAT-SC1 treatment and gastrectomy.

	R0 patients n=35 n (%)	R1–R2 patients n=15 n (%)	ITT population n=51 n (%)
Residual tumour classification			
R0			35 (68.6)
R1			2 (3.9)
R2			13 (25.5)
Missing tumour classification			1 (2.0)
Apoptosis (consensus)			
Patients evaluated	34	14	48
−	6 (17.6)	3 (21.4)	9 (18.8)
+	11 (32.4)	6 (42.9)	17 (35.4)
++	17 (50.0)	5 (35.7)	22 (45.8)
Tumour regression (consensus)			
Patients evaluated	31	13	44
0	11 (35.5)	7 (53.8)	18 (40.9)
+	6 (19.3)	5 (38.5)	11 (25.0)
++	14 (45.2)	1 (7.7)	15 (34.1)

ITT, intent to treat population.
